# The Impact of Injections of Different Nutrients on the Bacterial Community and Its Dechlorination Activity in Chloroethene-Contaminated Groundwater

**DOI:** 10.1264/jsme2.ME14127

**Published:** 2015-04-15

**Authors:** Takamasa Miura, Atsushi Yamazoe, Masako Ito, Shoko Ohji, Akira Hosoyama, Yoh Takahata, Nobuyuki Fujita

**Affiliations:** 1 Biological Resource Center, National Institute of Technology and Evaluation2–10–49 Nishihara, Tokyo 151–0066Japan; 2 Taisei Corporation344–1 Nase, Kanagawa 245–0051Japan

**Keywords:** bioremediation, dechlorination, chloroethene, *Dehalococcoides*, bacterial community

## Abstract

*Dehalococcoides* spp. are currently the only organisms known to completely reduce *cis*-1,2-dichloroethene (*cis*-DCE) and vinyl chloride (VC) to non-toxic ethene. However, the activation of fermenting bacteria that generate acetate, hydrogen, and CO_2_ is considered necessary to enhance the dechlorination activity of *Dehalococcoides* and enable the complete dechlorination of chloroethenes. In the present study, we stimulated chloroethene-contaminated groundwater by injecting different nutrients prepared from yeast extract or polylactate ester using a semicontinuous culture system. We then evaluated changes in the bacterial community structure and their relationship with dechlorination activity during the biostimulation. The populations of *Dehalococcoides* and the phyla *Bacteroidetes*, *Firmicutes*, and *Spirochaetes* increased in the yeast extract-amended cultures and chloroethenes were completely dechlorinated. However, the phylum *Proteobacteria* was dominant in polylactate ester-amended cultures, in which almost no *cis*-DCE and VC were dechlorinated. These results provide fundamental information regarding possible interactions among bacterial community members involved in the dechlorination process and support the design of successful biostimulation strategies.

Chloroethenes such as tetrachloroethene (PCE), trichloroethene (TCE), *cis*-1,2-dichloroethene (*cis*-DCE), and vinyl chloride (VC) are frequently detected as contaminants in groundwater, and are specified as environmentally regulated substances in Japan. Under anaerobic conditions, the biological reductive dechlorination of these chloroethenes to nontoxic ethene is catalyzed by dehalorespiring bacteria (26). While many bacteria transform PCE and TCE to *cis*-DCE, *Dehalococcoides* is the only organism known to be capable of dechlorinating *cis*-DCE and VC to ethene (19, 25). Accordingly, it is important that *Dehalococcoides* inhabits aquifers being remediated in order to enable the complete dechlorination of chloroethenes to ethene. Hendrickson *et al.* reported that a close relationship existed between the presence of the *Dehalococcoides* 16S rRNA gene and complete dechlorination in 24 samples with geographic origins scattered throughout North America and Europe (15).

Chloroethenes serve as the electron acceptor during anaerobic dechlorination, and electrons are derived from the oxidation of hydrogen and organic compounds such as acetate, lactate, pyruvate, butyrate, propionate, glucose, and alcohol (4, 6, 8, 9). Specifically, *Dehalococcoides* isolates exhibit low growth rates and specific essential nutrient requirements, including hydrogen as the electron donor, acetate as the carbon source, chlorinated or brominated compounds as respiratory electron acceptors, and vitamin B_12_ as a co-factor (20). Dechlorinating bacterial communities generally contain other microbes that are able to ferment organic substrates into acetate and hydrogen such as *Acetobacterium*, *Citrobacter*, *Clostridium*, *Desulfovibrio*, *Eubacterium*, and *Spirochaetes*, as well as *Dehalococcoides* as the key dechlorinating bacteria. Syntrophs such as *Desulfovibrio* and *Acetobacterium* have been shown to facilitate the growth and dechlorination activity of *Dehalococcoides mccartyi* 195^T^ through the fermentation of lactate to acetate and hydrogen and biosynthesis of vitamin B_12_ (14, 22). These finding suggest that the stimulation of syntrophs cohabiting with *Dehalococcoides* play a key role in efficient bioremediation.

Although Tang *et al.* reported that *D. mccartyi* 195^T^ contained complete amino acid biosynthesis pathways with acetate and CO_2_ as carbon sources (29), this strain has been shown to actively incorporate exogenous amino acids in order to facilitate growth and dechlorination (21), suggesting that an injection of amino acids direct enhances the dechlorination activity of *Dehalococcoides* inhabiting bioremediation sites.

In the present study, we demonstrated the biostimulation of anaerobic semicontinuous cultures using chloroethene-contaminated groundwater. Specifically, we investigated alterations in dechlorination activities, bacterial community structures, and *Dehalococcoides* densities in semicontinuous cultures injected with different nutrients that had been developed for groundwater remediation. Alternations in each of the above parameters were found to be closely related to the components of the nutrients. Our results provide useful information that will aid in the selection of proper nutrients for the biostimulation of chloroethenes degradation.

## Materials and Methods

### Construction of semicontinuous cultures

Groundwater (unconfined aquifer, GL –2.5 m to –11 m) and medium sand (GL –5.2 m to –5.4 m) were collected from a chloroethene-contaminated aquifer in Japan. The base semicontinuous culture was constructed by mixing 550 mL of groundwater, 20 g of medium sand, and 3 mL of TCE solution (70 mg L^−1^; Kanto Chemicals, Tokyo, Japan) in a 500-mL culture bottle. The nutrients, HRC (Regenesis, San Clemente, CA, USA), TM-B (Taisei, Kanagawa, Japan), and EDC (Ecocycle, Tokyo, Japan) were diluted 10-fold in deionized water, after which 2.8-mL aliquots were added to the base semicontinuous cultures. To achieve pH control, 1.7 mL of NaHCO_3_^(^75 g L^−1^) and NaCO_3_ (25 g L^−1^) were added to the culture injected with EDC. These cultures were then grown in a continuously stirred chamber at 20°C for a total of 115 d.

### Analytical procedures

Chlorinated compounds were analyzed by gas chromatography/mass spectrometry (Network GC System; Agilent Technologies, Palo Alto, CA, USA) through a headspace sampler (200°C upon injection, Network Headspace Sampler; Agilent Technologies) and a GC column (HP-1, 60 m×0.32 mm×1.0 μm; Agilent Technologies). The column was held at 35°C for 2 min, after which the temperature was increased to 170°C at 15°C min^−1^. Helium was applied as the carrier gas at a flow rate of 1.5 mL min^−1^. Ions were analyzed using a HPLC system (Alliance 2695XC Separation Module; Waters, Milford, MA, USA). Cations were analyzed using a Shodex IC YS-50 column (Showa Denko, Tokyo, Japan) with CH_3_SO_3_H (4 mM) applied at a flow rate of 1.0 mL min^−1^ and a temperature of 40°C. Anions were analyzed using a Shodex IC SI-50 4E column (Showa Denko) with Na_2_CO_3_ (3.2 mM) and NaHCO_3_ (1.0 mM) at a flow rate of 0.8 mL min^−1^ and a temperature of 40°C. Dissolved organic carbon (DOC) was analyzed using a total organic carbon analyzer (TOC-5000A; Shimadzu, Kyoto, Japan) after filtering samples through 0.45-μm pore size poly-ethersulfone membranes (Millipore, Tokyo, Japan). Oxidation-reduction potential (ORP) and pH were measured using a Horiba pH meter D-24 and D-52 (Horiba, Kyoto, Japan), respectively. The total number of bacteria was determined by the acridine orange direct count method (16).

### DNA extraction

Each 5-mL culture was filtered through a 0.22-μm pore size poly-carbonate membrane (Millipore) and then stored at −80°C until later analyses. The biomass was then suspended in 567 μL of TE buffer (pH 8.0), 30 μL of sodium dodecyl sulfate (10% [w/v]), and 3 μL of proteinase K (20 mg mL^−1^), after which the mixture was incubated at 50°C for 60 min. A total of 100 μL of NaCl (5 M) and 80 μL of cetyltrimethylammonium bromide (10% [w/v])/NaCl (0.7 M) solution were then added to the mixture, which was incubated at 65°C for 10 min. Eight hundred microliters of chloroform/isoamyl alcohol (24:1) was then added and mixed gently, after which the mixture was centrifuged for 5 min at room temperature and 20,000×*g*. A total of 600 μL of supernatant was then recovered and an equal amount of phenol/chloroform/isoamyl alcohol (25:24:1 [v/v/v]) was added. After mixing gently, the mixture was centrifuged for 5 min at room temperature and 20,000×*g*, and 550 μL of supernatant was recovered. A total of 5.5 μL of the precipitation carrier (Takara Bio, Otsu, Japan), 55 μL of sodium acetate (3 M, pH5.2), and 550 μL of isopropyl alcohol were then added and the mixture was centrifuged for 10 min at 4°C and 20,000×*g*. The DNA pellet was then washed with 1 mL of ethanol (70% [v/v]) and centrifuged at 4°C and 20,000×*g* for 5 min. The air-dried DNA pellet was dissolved in 50 μL of TE buffer.

### Quantitative PCR

The copy number of *Dehalococcoides* 16S rRNA genes was determined by quantitative PCR using an Applied Biosystems 7500 Fast Real-Time PCR System (Life Technologies, Gaithersburg, MD, USA). The primers, 624-Fw (5′-CAGCAGGAGAAAACGGAATT-3′) and 1232-Rv (5′-GACAGCTTTGGGGATTAGC-3′), have been described previously (30). Each 25-μL reaction mixture contained 10.8 μL of sterilized deionized water, 12.5 μL 2×SYBR Premix (Takara Bio), 0.1 μL 624-Fw primer (100 μM), 0.1 μL 1232-Rv primers (100 μM), 0.5 μL 50×ROX Dye (Takara Bio), and 1 μL extracted DNA. PCR was conducted by subjecting the samples to 95°C for 30 s, followed by 40 cycles of 95°C for 5 s, 54°C for 30 s, and 72°C for 45 s.

### 16S rRNA gene amplicon sequencing and data analysis

The 16S rRNA genes were amplified by PCR using the primers 338F_PA_V3F (5′-CGTATCGCCTCCCTCGCGCCATCAGACTCCTACGGGAGGCAGCAG-3′) (17) and 1046R_PB_V6R (5′-CTATGCGCCTTGCCAGCCCGCTCAGCGACAGCCATGCAGCACCT-3′) (27) containing adaptor and key sequences (underlines) for 454 GS FLX Titanium pyrosequencing (Roche, Basel, Switzerland). Each 25-μL reaction mixture contained 2.5 μL 10×buffer, 2.5 μL dNTPs (2 mM each), 1 μL DMSO, 1 μL 0.1% bovine serum albumin, 1 μL of each primer (10 μM), 0.25 μL Blend Taq (2.5 U μL^−1^) (Toyobo, Osaka, Japan), and 1 μL template DNA (approximately 3 ng). The cycling conditions were as follows: 95°C for 5 min followed by 23 cycles of 94°C for 1 min, 50°C for 30 s, and 68°C for 1 min, and then a final extension at 70°C for 10 min. PCR products were purified using the Wizard SV Gel and PCR Clean-Up System (Promega, Madison, WI, USA). The concentration of PCR products was estimated using PicoGreen dsDNA quantitation reagent (Molecular Probes, Karlsruhe, Germany), and the quality of PCR products was checked using an Agilent 2100 Bioanalyzer (Agilent Technologies). The amplicon samples were sequenced using a GS FLX Titanium system (Roche). Bacterial community analyses based on the 16S rRNA gene and a principal coordinate analysis (PCoA) were performed using the MacQIIME software package (2) and Greengenes 13_5 dataset (5). A phylogenetic tree of *Dehalococcoides* was constructed by the maximum likelihood estimation method using MEGA software version 5.2 (28). Denoised and trimmed sequences were deposited in the DDBJ Sequence Read Archive under accession numbers DRA002417 (control_day115, day13, day40, day73 and day88; DRR021760 to DRR021764, HRC_day115, day13, day40, day73 and day88; DDR021750 to DDR021754, TM-B_day115, day13, day40, day73 and day88; DRR021755 to DRR021759, EDC_day115, day13, day40, day73 and day88; DRR021745 to DRR021749).

## Results

### Analysis of key parameters after the nutrient treatment

To enhance the anaerobic dechlorination activity of the dechlorinating organisms in the groundwater, the nutrients HRC, TM-B, and EDC were injected into semicontinuous cultures. HRC was mainly composed of poly-lactate ester to produce the controlled release of lactic acid, while TM-B and EDC were derived from yeast extract (100% and 19%, respectively). In addition, EDC contained lactose (65%) and sodium propionate (15%) (U.S. Patent No. 8,790,912). A semicontinuous culture without nutrients was used as a negative control.

The groundwater used in this study was contaminated with 466 μL^−1^ of *cis*-DCE and 197 μg L^−1^ of PCE, while the levels of VC and TCE were low (47 μg L^−1^ and 39 μg L^−1^, respectively) (Table 1). The pH and oxidation-reduction potential (ORP) values of groundwater before the injection of the nutrients were 6.3 and −180 mV, respectively, and the DOC was only 4.4 mg L^−1^. Although the total number of bacteria was 3.1×10^6^, only a small amount of *Dehalococcoides* was present (4.5 cells mL^−1^).

To evaluate the effects of the nutrient injection, semicontinuous cultures were monitored between 0 and 115 d during eight incubation periods (Table 2). The ORP was +134 mV at 0 d (Table 2), while this value ranged from −164 mV to −277 mV in cultures injected with nutrients during the incubation period, indicating that the conditions remained anaerobic throughout the experiment (115 d). The DOC of cultures treated with TM-B and EDC were 146.7 and 203.1 mg L^−1^ at 0 d, while these values decreased to 12.0 and 9.4 mg L^−1^ at 115 d, respectively (Table 2). However, reductions in the DOC of the culture with HRC occurred slightly later (from 201.4 mg L^−1^ [0 d] to 57.0 mg L^−1^ [115 d]). Although the pH of the cultures treated with TM-B and EDC remained at approximately 6.8, that of the culture treated with HRC decreased to below 6.4.

The ammoniacal nitrogen (NH_4_-N) of the cultures treated with TM-B and EDC increased from 2.6 mg L^−1^ and ND (not detected) at 0 d to 26.4 mg L^−1^ and 3.9 mg L^−1^ at 13 d, respectively. However, the NH_4_-N of the control culture and the cultures treated with HRC was less than 1.0 mg L^−1^. Phosphate phosphorus (PO_4_-P) was detected in the cultures treated with TM-B at 18.8 mg L^−1^ at 0 d, but was absent at 115 d. SO_4_^2−^ was detected in all cultures at levels ranging from 57.3 to 59.5 mg L^−1^ at 0 d, but was reduced to 0.9 mg L^−1^ and 2.6 mg L^−1^ after 13 d in cultures treated with TM-B and EDC, respectively. Additionally, SO_4_^2−^ decreased from 45.5 mg L^−1^ at 13 d to ND at 27 d in cultures treated with HRC.

### Monitoring of chloroethenes and *Dehalococcoides* population

**Fig. 1:**
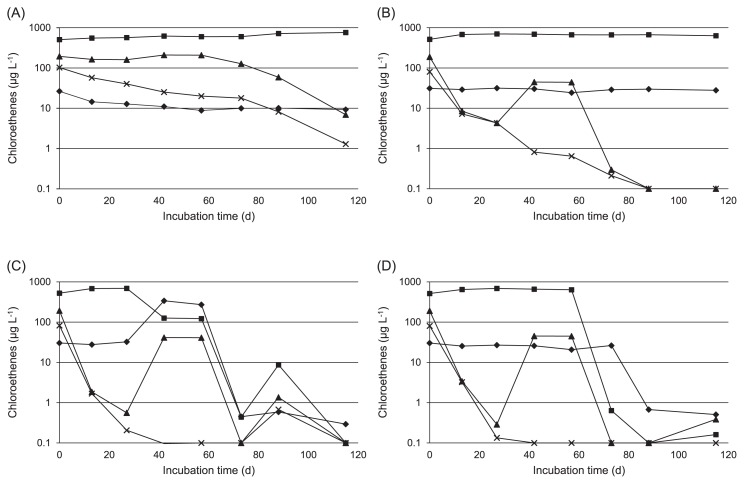
Dechlorination of chloroethenes in control culture (A), cultures with HRC (B), TM-B (C), and EDC (D). Crosses, PCE; filled triangles, TCE; filled squares, *cis*-DCE; filled diamonds, VC.

The time course of chloroethene levels in the cultures is shown in Fig. 1. PCE and TCE slowly decreased in the control culture and the culture treated with HRC; however, significant reductions in *cis*-DCE and VC were not confirmed (Fig. 1A and B).

PCE and TCE decreased more rapidly in cultures treated with TM-B and EDC than in the control culture and the culture treated with HRC, then continued to decrease until they were near the detection limit within 20 d (Fig. 1C and D). In the culture treated with TM-B, *cis*-DCE decreased from 689.6 μg L^−1^ at 27 d to 126.4 μg L^−1^ at 42 d, while VC increased from 27.8 μg L^−1^ at 13 d to 338.8 μg L^−1^ at 42 d (Fig. 1C). However, *cis*-DCE and VC then decreased to 0.5 μg L^−1^ and 0.4 μg L^−1^ at 73 d, respectively. Although *cis*-DCE remained almost constant for 57 d (637.5 μg L^−1^) in the culture treated with EDC, it decreased to 0.6 μg L^−1^ at 73 d (Fig. 1D). Similarly, VC remained relatively high until day 73 (26.3 μg L^−1^), but decreased to 0.7 μg L^−1^ by 88 d.

**Fig. 2:**
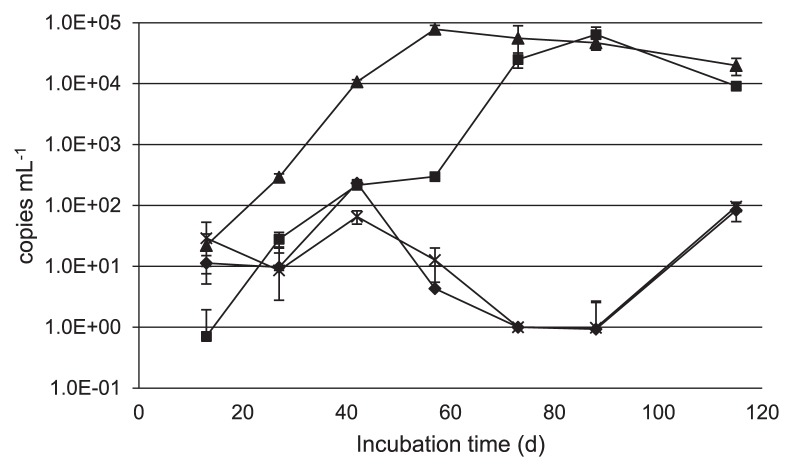
The number of *Dehalococcoides* cells in semicontinuous cultures. Crosses, culture control; filled diamonds, culture HRC; filled triangles, culture TM-B; filled squares, culture EDC. Error bars indicate standard deviations (*n*=3).

The cell density of *Dehalococcoides* in each culture was measured by quantitative real-time PCR targeting the 16S rRNA gene copy number of *Dehalococcoides* (Fig. 2). The cell density of the culture treated with TM-B increased from 2.2±1.2×10 cells mL^−1^ at 13 d to 7.8±1.2×10^4^ cells mL^−1^ at 57 d, then gradually decreased to 2.0±0.6×10^4^ cells mL^−1^ at 115 d. Similarly, the cell density of the culture treated with EDC in-creased from 0.7±1.2 cells mL^−1^ at 13 d to 6.4±2.1×10^4^ cells mL^−1^ at 88 d, then decreased to 9.1±0.3×10^3^ cells mL^−1^ at 115 d. Although *Dehalococcoides* increased slightly at 40 and 115 d in the control culture and the culture treated with HRC, it showed almost no increase throughout the incubation periods. These changes in the cell densities of *Dehalococcoides* matched the dechlorination activities of the respective cultures.

### Analysis of the bacterial community by 16S rRNA gene amplicon sequencing

**Fig. 3:**
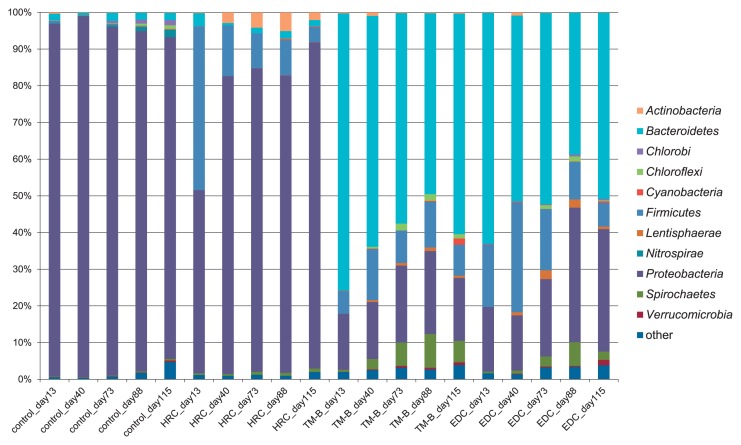
Bacterial diversity of semicontinuous cultures at the phylum level.

We conducted 16S rRNA gene amplicon sequencing to investigate bacterial communities during the dechlorination process in semicontinuous cultures. A total of 259,715 sequences were obtained after denoising and removing the chimeric sequences. A taxonomic analysis at the phylum level is shown in Fig. 3, while a taxonomic analysis at the genus level of the major taxonomic groups, which accounted for more than 1% in at least one culture, is shown in Table 3.

In the control culture, *Proteobacteria* was dominant on all incubation days (96.2% at 13 d, 98.4% at 40 d, 95.0% at 73 d, 92.8% at 88 d, and 87.7% at 115 d), and the class *Betaproteobacteria* accounted for over half of the *Proteobacteria* (66.1%, 60.7%, 66.9%, 64.0%, and 66.7%, respectively). These results demonstrated that the phylum *Proteobacteria* was dominant in the groundwater used in this study. At the genus level, *Sphingomonas*, *Janthinobacterium*, and *Pseudomonas* accounted for up to 95% of the community (Table 3), while almost no members of the classes *Deltaproteobacteria* and *Epsilonproteobacteria* were observed.

In the culture treated with HRC, the bacterial community structure showed some similarity to that of the control culture, although the level of the members of the phylum *Firmicutes* was higher, reaching 44.7% at 13 d. The OTUs assigned to the genus unassigned 5 (family *Veillonellaceae*, Table 3) and the genus *Pelosinus* accounted for 12.9% and 24.4% of the population, respectively. Furthermore, levels of the genus *Propionicimonas* (phylum *Actinobacteria*) increased from 0.0% at 13 d to 5.1% at 88 d, and the proportions of the genus *Anaeromusa* (phylum *Clostridium*) were greater than 2.2% from 0 to 88 d.

In the culture treated with TM-B, the proportion of the phylum *Bacteroidetes* decreased from 75.7% at 13 d to 49.3% at 88 d, then increased to 60.1% at 115 d. The OTU assigned to the genus unassigned 2 (family *Porphyromonadaceae*, Table 3) was the most abundant genus in *Bacteroidetes* at 13 d; however, the OTU gradually decreased to 20.5% at 115 d. Among *Proteobacteria*, *Gammaproteobacteria* was the most abundant class at 13 d, while *Deltaproteobacteria* containing the genera *Desulfovibrio* and *Geobacter* and the family *Syntrophorhabdaceae* gradually increased from 7.3% of the *Proteobacteria* at 13 d to 48.6% at 88 d. Concomitantly, *Dehalococcoides* increased from 0% at 13 d to 1.7% at 88 d (1.8% at 73 d), while the genus *Treponema* (phylum *Spirochaetes*) increased from 0.6% at 13 d to 9.2% at 88 d.

In the culture treated with EDC, *Dehalococcoides* and *Treponema* showed slower increases than those in the culture treated with TM-B. Specifically, the levels of these organisms increased from 0% at 13 d to 1.3% at 88 d and from 0.4% at 13 d to 6.5% at 88 d, respectively. The proportion of the syntroph *Desulfovibrio* (1.1% at 73 d) was also smaller than that in the culture treated with TM-B (2.6% at 73 d). The alternations observed in *Dehalococcoides* and these bacteria in the cultures treated with TM-B and EDC coincided closely with the dechlorination of chloroethenes.

**Fig. 4:**
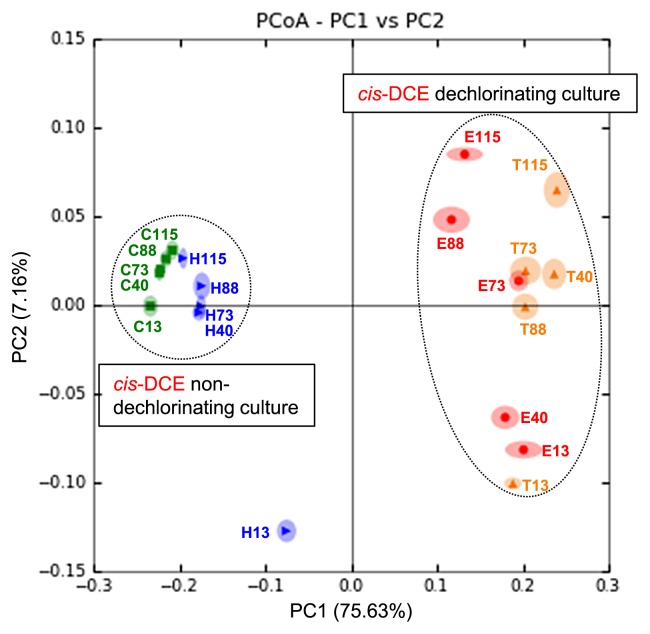
Principal coordinate analysis (PCoA) using the MacQIIME pipeline. C13; control_day13, C40; control_day40, C73; control_day73, C88; control_day88, C115; control_day115, H13; HRC_day13, H40; HRC_day40, H73; HRC_day73, H88; HRC_day88, H115; HRC_day115, T13; TM-B_day13, T40; TM-B_day40, T73; TM-B_day73, T88; TM-B_day88, T115; TM-B_day115, E13; EDC_day13, E40; EDC_day40, E73; EDC_day73, E88; EDC_day88, E115; EDC_day115.

A PCoA analysis confirmed that the bacterial community structures and their temporal variations were highly dependent on nutrient treatments (Fig. 4). The first principal coordinate axis (PC1) and second principal coordinate axis (PC2) accounted for 75.63% and 7.16% of the overall variation, respectively. The structures of the cultures treated with TM-B and EDC (*cis*-DCE dechlorinating cultures), as well as those of the control culture and the culture treated with HRC (*cis*-DCE non-dechlorinating cultures) were highly similar. Moreover, temporal variations, which were well represented by PC2 in each culture, were highly synchronous between cultures treated with TM-B and EDC, and between the control culture and the culture treated with HRC, respectively. The only exception was the culture treated with HRC at 13 d (Fig. 4), for which a unique abundance of the genus *Pelosinus (*24.4%) and *Veillonellaceae* bacteria (12.9%) was observed (Table 3). Men *et al.* also reported that bacteria related to *Pelosinus* spp. were the most abundant species in a dechlorinating culture amended with lactate (23). The reason for the failure of these bacteria to continue to grow beyond 13 d under our experimental conditions currently remains unclear.

### Phylogenetic analysis of *Dehalococcoides* in semicontinuous cultures

**Fig. 5:**
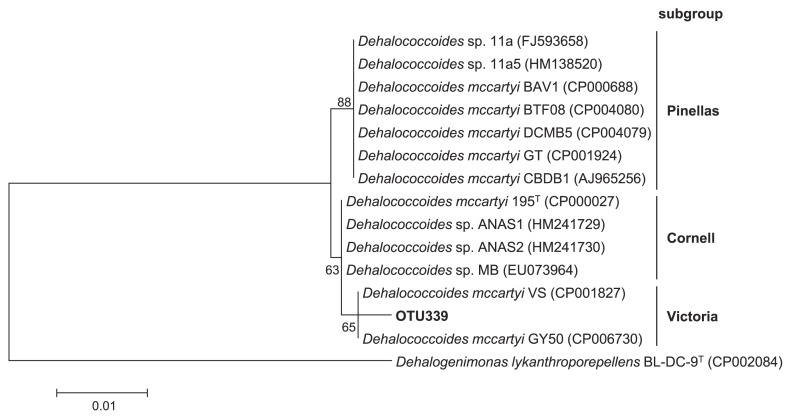
Phylogenetic analysis of *Dehalococcoides* strains and OTU339 based on partial 16S rRNA gene sequences. The tree was calculated using a maximum-likelihood estimation with bootstrap values. *Dehalogenimonas lykanthroporepellens* BL-DC-9^T^ was used as an outgroup.

The 16S rRNA genes of the *Dehalococcoides* isolates share >98% similarity with each other (20). Therefore, only one major OTU (OTU339) assigned to the genus *Dehalococcoides* was generated by an analysis of the QIIME pipeline using a 97% similarity threshold. The phylogenetic analysis revealed that the representative sequence of this OTU was affiliated with the Victoria subgroup of *Dehalococcoides* and showed 99.5% (550/553) similarity to *D. mccartyi* VS (Fig. 5).

## Discussion

In the present study, we demonstrated the anaerobic biostimulation of semicontinuous cultures using chloroethene-contaminated groundwater and compared reductive dechlorination in response to three nutrient additives. The initial concentration of *cis*-DCE in the groundwater sample used in this study was higher (446 μg L^−1^) than that of other chloroethene species (Table 1). Since the cell density of indigenous *Dehalococcoides* was very low (4.5 cells mL^−1^), natural attenuation by anaerobic dechlorination from *cis*-DCE to VC appeared to have occurred slowly. In the control culture, the cell density of *Dehalococcoides* was decreased with a concomitant elevation in the ORP from 42 to 73 d; however, the primary cause of this elevation has yet to be clarified (Table 2 and Fig. 2). After being incubated for 115 d, PCE and TCE were completely dechlorinated to *cis*-DCE in all cultures injected with nutrients, even though the *Dehalococcoides* population was low throughout the incubation period (Table 3). These results suggested that dehalorespiring bacteria other than *Dehalococcoides* were primarily responsible for the transformation of PCE and TCE to *cis*-DCE. While cultures treated with TM-B and EDC actively dechlorinated *cis*-DCE and VC to ethene, *cis*-DCE and VC accumulated in the culture treated with HRC, despite the ORP remaining at approximately −220 mV from 13 to 115 d (Table 2). This may have been causally related, at least in part, to the pH of the culture treated with HRC, which was below 6.4 from 0 to 115 d. It was assumed that the activity of *Dehalococcoides* was low in the culture treated with HRC because the dechlorination and growth of *Dehalococcoides* preferentially occurred at a neutral pH of 6.0–8.0, with the highest activity occurring between pH 6.9 and 7.5 (20). The cell density of the culture treated with HRC decreased from 42 to 88 d with a concomitant decrease in pH (Table 2 and Fig. 2). Alternatively, because the DOC in the culture injected with HRC decreased at a slower rate than in those treated with TM-B or EDC (Table 2), the available concentration of organic carbons such as acetate derived from polylactate ester may not have been sufficient to enhance the growth and dechlorination activity of *Dehalococcoides*. The NH_4_-N levels of the culture treated with TM-B were 5-fold greater than those of the culture treated with EDC, whereas no NH_4_-N was generated in the control culture or the culture treated with HRC (Table 2). NH_4_-N is considered to be a degraded product derived from the yeast extract present in the nutrients; therefore, the amino acids required for the activation of *Dehalococcoides* were in short supply in the culture treated with HRC.

Previous studies have also shown that *cis*-DCE and VC were dechlorinated in a culture injected with EDC (18), but not in cultures injected with HRC or lactate (6, 24). Taken together, these findings suggested that these differential effects were the result of differences in the components of each nutrient rather than or in addition to differences in pH or the overall availability of carbon sources. TM-B and EDC contained 100% and 19% yeast extract, respectively, while the main component of HRC is polylactate, suggesting that *Dehalococcoides* prefers components such as amino acids and inorganic materials present in yeast extract. The importance of amino acids was also supported by the previous finding that *D. mccartyi* 195^T^ not only had the potential to synthesize all amino acids, but also strongly imported some amino acids including phenylalanine, isoleucine, leucine, and methionine in laboratory cultures (31). Supplementation with amino acids is thought to be an important factor for enhancing the dechlorination activity of *Dehalococcoides* during the *in situ* bioremediation of chloroethene-contaminated aquifers if the aquifer lacks these amino acids.

Bacterial communities containing other dechlorinaters and syntrophs are also considered to exert a stimulatory effect on *Dehalococcoides*. The bacterial community structures of cultures treated with TM-B and EDC were similar (Fig. 3), and the cell densities of *Dehalococcoides* increased to 7.8±1.2×10^4^ cells mL^−1^ and 6.4±2.1×10^4^ cells mL^−1^, respectively (Fig. 2). Previous studies reported that *Dehalococcoides* was often found with PCE and TCE degraders such as *Dehalobacter*, *Desulfitobacterium*, *Geobacter*, and *Sulfurospirillum* (1, 3, 6, 7, 25). Of these dechlorinating bacteria, *Geobacter* occurred most frequently in this study, being present in all cultures (Table 3). This was followed by *Dehalobacter* and *Sulfurospirillum*, which were present in smaller amounts in cultures treated with TM-B and EDC (data not shown). Conversely, although *Desulfovibrio*, a known syntroph, was found in the control culture and the culture treated with HRC (Table 3), *cis*-DCE and VC remained almost unchanged in these cultures, indicating that the dechlorination activity of *Dehalococcoides* was not enhanced by this syntroph. These results suggest that suitable conditions for syntrophs are necessary for the activation of *Dehalococcoides*. The population of the genus *Treponema (*phylum *Spirochaetes*) increased simultaneously with *Dehalococcoides* from 13 to 88 d in cultures treated with TM-B and EDC (Fig. 3 and Table 3), and this genus was also detected in other dechlorination cultures (7, 10, 11, 13). This genus is a homoacetogen that is able to ferment some carbohydrates to acetate, hydrogen, and CO_2_, which are essential compounds for the growth of *Dehalococcoides* (12). Thus, *Treponema* may be the key syntroph required for the dechlorination of *cis*-DCE and VC by *Dehalococcoides*.

In a previous study, the complete dechlorination of TCE was observed in a dechlorinating consortium (ANAS) injected with lactate, in which the known fermenters of lactate and the other simple organics *Bacteroides*, *Clostridium*, and *Citrobacter*, were present (8). In this culture, high concentrations of hydrogen were rapidly generated, while dechlorination to ethene decreased simultaneously. Although *Clostridium* was detected in the present study, almost no *Bacteroides* or *Citrobacter* were detected in the culture treated with HRC (Fig. 3 and Table 3). These results suggest that *Bacteroides* and *Citrobacter* are important syntrophs for *Dehalococcoides* when fed with lactate.

In conclusion, we herein demonstrated that injections of TM-B and EDC increased the *Dehalococcoides* population and enhanced the reductive dechlorination of chloroethenes to ethene in this chloroethene-contaminated groundwater, possibly via the supplementation of compounds contained in yeast extract such as amino acids. Furthermore, the enhanced growth and dechlorination activity of *Dehalococcoides* coincided with increased proportions of the phyla *Bacteroidetes*, *Firmicutes*, and *Spirochaetes* in the culture. These results provide useful information regarding the choice of appropriate nutrients and microorganisms as indicators for efficient biostimulation.

## Figures and Tables

**Table 1:** Parameter analysis of the groundwater used for semicontinuous cultures

Chloroethenes (μg L^−1^)	
tetrachloroethene	197
trichloroethene	39
*cis*-1,2-dichloroethene	466
*trans*-1,2-dichloroethene	2
1,1-dichloroethene	1
vinyl chloride	47

pH	6.3
ORP (mV)	−180
DOC (mg L^−1^)	4.4

Total number of bacteria (cells mL^−1^)	3.1×10^6^
Number of *Dehalococcoides* (cells mL^−1^)	4.5

**Table 2:** Parameter analysis of semicontinuous cultures

Incubation time (d)	ORP (mV)				DOC (mg L^−1^)				pH			
		
	control	HRC	TM-B	EDC	control	HRC	TM-B	EDC	control	HRC	TM-B	EDC
0	134	134	134	134	5.7	201.4	146.7	203.1	6.4	6.4	6.5	7.0
13	74	−217	−230	−231	3.2	183.3	101.9	147.4	6.5	5.9	6.5	6.3
27	−176	−219	−182	−182	3.6	156.5	73.6	136.6	6.8	6.3	6.6	6.4
42	6	−236	−187	−249	3.2	133.1	41.4	117.2	6.6	5.6	6.8	6.4
57	196	−243	−277	−267	1.3	160.1	43.7	85.9	6.4	5.6	6.5	6.7
73	201	−209	−190	−181	2.9	113.4	26.6	36.5	6.9	6.1	7.0	7.0
88	−170	−195	−164	−185	2.3	86.4	14.0	24.1	6.8	6.1	6.8	6.8
115	−178	−216	−214	−168	1.7	57.0	12.0	9.4	6.7	6.3	6.8	6.9

Incubation time (d)	NH_4_-N (mg L^−1^)				PO_4_-P (mg L^−1^)				SO_4_^2−^ (mg L^−1^)			
		
	control	HRC	TM-B	EDC	control	HRC	TM-B	EDC	control	HRC	TM-B	EDC

0	1.0	1.0	2.6	ND	ND	ND	18.8	ND	57.9	57.3	57.8	59.5
13	0.9	ND	26.4	3.9	ND	ND	14.1	ND	58.0	45.5	0.9	2.6
27	0.9	ND	27.0	4.8	ND	ND	7.9	ND	56.6	ND	0.6	ND
42	1.0	ND	26.8	5.1	ND	ND	6.4	ND	57.0	0.7	ND	0.7
57	0.8	ND	25.9	4.8	ND	ND	5.9	ND	56.4	0.7	0.6	0.6
73	0.9	ND	24.8	4.9	ND	ND	8.4	ND	56.2	1.1	ND	ND
88	1.0	ND	23.3	4.9	ND	ND	8.6	ND	56.3	0.5	0.6	0.4
115	1.0	ND	22.3	4.7	ND	ND	ND	ND	57.5	ND	8.7	ND

ND, not detected

**Table 3:** Major taxonomic groups found by 16S rRNA gene amplicon sequencing (%)

phylum	order	genus	control					HRC				
	
			day 13	day 40	day 73	day 88	day 115	day 13	day 40	day 73	day 88	day 115
*Actinobacteria*	*Actinomycetales*	*Propionicimonas*	<0.1	<0.1	ND	ND	ND	ND	2.8	4.2	5.1	2.0
*Bacteroidetes*	*Bacteroidales*	*Bacteroides*	ND	<0.1	<0.1	<0.1	<0.1	ND	ND	<0.1	ND	<0.1
		Blvii28	0.1	<0.1	<0.1	<0.1	0.1	0.2	<0.1	0.1	0.1	ND
		unassigned 1	1.1	0.4	1.7	1.6	0.9	3.0	0.2	1.1	1.8	1.3
		unassigned 2	0.2	<0.1	0.1	<0.1	<0.1	0.3	<0.1	ND	ND	0.1
*Chloroflexi*	*Dehalococcoidales*	*Dehalococcoides*	<0.1	ND	ND	<0.1	ND	ND	ND	ND	ND	ND
*Firmicutes*	*Bacilli*	unassigned 3	<0.1	<0.1	<0.1	ND	ND	0.1	<0.1	ND	ND	ND
	*Clostridiales*	*Anaeromusa*	ND	ND	ND	ND	ND	2.2	8.9	6.3	4.0	0.6
		*Pelosinus*	<0.1	ND	ND	ND	ND	24.4	0.6	0.3	0.5	0.2
		unassigned 4	<0.1	ND	<0.1	ND	ND	<0.1	0.1	<0.1	0.6	0.2
		unassigned 5	<0.1	ND	ND	<0.1	<0.1	12.9	0.2	0.7	1.1	0.3
*Proteobacteria*	*Sphingomonadales*	*Sphingomonas*	5.8	10.1	13.5	16.0	11.1	0.7	10.0	10.5	10.3	13.9
	*Burkholderiales*	*Janthinobacterium*	28.5	58.1	59.3	55.9	50.7	2.9	44.7	52.6	49.6	55.2
	*Desulfovibrionales*	*Desulfovibrio*	<0.1	<0.1	<0.1	<0.1	0.1	4.8	0.7	2.5	3.4	1.6
	*Desulfuromonadales*	*Geobacter*	1.7	0.3	0.8	1.2	2.6	6.6	0.5	2.5	5.2	1.6
	*Pseudomonadales*	*Pseudomonas*	7.3	26.9	13.8	12.3	10.8	4.6	24.6	13.0	10.6	15.5
*Spirochaetes*	*Spirochaetales*	*Treponema*	0.2	0.1	0.2	0.2	0.4	0.5	0.5	0.8	0.7	0.7

phylum	order	genus	TM-B					EDC				
	
			day 13	day 40	day 73	day 88	day 115	day 13	day 40	day 73	day 88	day 115

*Actinobacteria*	*Actinomycetales*	*Propionicimonas*	ND	<0.1	<0.1	<0.1	ND	0.1	0.5	0.1	<0.1	<0.1
*Bacteroidetes*	*Bacteroidales*	*Bacteroides*	1.5	4.3	4.8	5.0	5.5	1.7	2.9	1.6	0.6	3.9
		Blvii28	2.4	1.1	3.0	2.0	4.7	5.6	3.6	5.9	2.3	20.0
		unassigned 1	1.4	26.1	16.7	11.5	27.9	7.6	11.6	19.9	16.4	17.7
		unassigned 2	62.0	29.1	31.3	29.1	20.5	45.9	31.4	23.6	18.9	8.1
*Chloroflexi*	*Dehalococcoidales*	*Dehalococcoides*	ND	0.5	1.8	1.7	0.9	ND	<0.1	0.9	1.3	0.2
*Firmicutes*	*Bacilli*	unassigned 3	0.2	1.8	0.6	0.5	1.6	9.2	15.4	9.2	3.8	3.4
	*Clostridiales*	*Anaeromusa*	ND	ND	<0.1	<0.1	<0.1	ND	ND	ND	ND	ND
		*Pelosinus*	<0.1	0.1	<0.1	<0.1	<0.1	0.2	0.1	ND	0.1	<0.1
		unassigned 4	0.1	1.2	1.3	1.5	1.2	0.3	0.9	1.6	1.4	0.7
		unassigned 5	0.1	0.3	0.7	0.6	0.5	0.3	0.2	0.4	0.2	0.5
*Proteobacteria*	*Sphingomonadales*	*Sphingomonas*	0.1	1.5	1.7	1.7	1.5	0.7	1.3	2.5	6.1	3.6
	*Burkholderiales*	*Janthinobacterium*	0.9	7.1	8.9	6.5	5.4	2.1	7.5	8.9	17.4	18.7
	*Desulfovibrionales*	*Desulfovibrio*	0.7	0.8	2.6	2.2	1.7	0.7	0.4	1.1	0.9	0.8
	*Desulfuromonadales*	*Geobacter*	0.4	0.9	1.1	1.2	0.4	0.5	0.8	1.9	2.4	1.1
	*Pseudomonadales*	*Pseudomonas*	2.4	3.4	2.4	1.7	1.6	0.7	3.9	2.4	4.5	4.0
*Spirochaetes*	*Spirochaetales*	*Treponema*	0.6	2.8	6.4	9.2	5.9	0.4	0.8	2.7	6.5	2.3

ND, not detected
